# Knowledge, Attitude, and Practices toward Hepatitis B Infection among Healthcare Students—A Nationwide Cross-Sectional Study in Jordan

**DOI:** 10.3390/ijerph20054348

**Published:** 2023-02-28

**Authors:** Nader Alaridah, Rayan M. Joudeh, Haneen Al-Abdallat, Raba’a F. Jarrar, Layan Ismail, Mohammad Jum’ah, Zaina Alnajjar, Eman Alzyoud, Zaina Battah, Aya Battah, Manar Alshami, Anas H. A. Abu-Humaidan

**Affiliations:** 1 Department of Pathology, Microbiology, and Forensic Medicine, School of Medicine, The University of Jordan, Amman 11942, Jordan; 2 College of Medicine, Sulaiman Al-Rajhi University, Al-Bukayriah 52726, Saudi Arabia; 3 School of Medicine, The University of Jordan, Amman 11942, Jordan; 4 Department of the Clinical Laboratory Sciences, School of Science, The University of Jordan, Amman 11942, Jordan; 5 Faculty of Medicine, Al-Balqa Applied University, As-Salt 19117, Jordan; 6 Faculty of Medicine, Hashemite University, Zarqa 13116, Jordan; 7 Ministry of Health, Amman 11118, Jordan; 8 School of Pharmacy, The University of Jordan, Amman 11942, Jordan; 9 School of Dentistry, The University of Jordan, Amman 11942, Jordan; 10 Faculty of Medicine, Yarmouk University, Irbid 21163, Jordan

**Keywords:** hepatitis B virus, knowledge, attitude, practice, Jordan

## Abstract

The World Health Organization has estimated that around 66 thousand HBV infection cases are caused by needlestick injuries annually. Healthcare students should be aware of HBV transmission routes and preventive measures. This study assessed the knowledge, attitudes, and practices toward HBV among Jordanian healthcare students and its associated factors. A cross-national study was conducted from March to August 2022. The questionnaire was composed of four sections: participants’ sociodemographics, knowledge, attitudes, and practices about HBV, and 2322 participants were enrolled. The collected responses were analyzed with SPSS software (version 25 (IBM Corp., Armonk, NY, USA)) using descriptive statistics, unpaired t-tests, chi-square tests, and multivariate regression analyses. A *p*-value ≤ 0.05 was considered statistically significant. The results showed that 67.9% were females, 26.4% were medical students, and 35.9% were in their 3rd year. Overall, 40% of the participants held high levels of knowledge and attitudes. Further, 63.9% of participants had good practices toward HBV. Gender, year of study, encountering HBV patients, college, and having extra HBV courses were associated with high levels of KAP. This study demonstrated insufficient knowledge and attitudes toward HBV; however, the practice level toward HBV among healthcare students was promising. Therefore, public health efforts should modify the knowledge and attitude gaps to reinforce awareness and minimize the risk of infection.

## 1. Introduction

Hepatitis B virus (HBV) infection is a worldwide public health problem, and it is considered the most significant global risk factor for liver cancer. In 2019, The World Health Organization (WHO) reported around 296 million people were living with the virus globally [1]. The prevalence of HBV in the Jordanian general population has dropped from 9.9% in 1985 to 2.4% in 2016 after the introduction of the HBV vaccination program to the newborn and high-risk groups [2]. HBV is transmitted through direct contact with infected body fluids, such as blood, semen, mucous membranes, saliva, unsafe injections, and from an infected mother to her child at birth (perinatal transmission) [3]. The Centers for Disease Control and Prevention (CDC) recommended HBV vaccination for healthcare workers, as they are at risk for needle stick injury and vulnerable to contact with infected blood and body fluids [4]. A meta-analysis study showed the incidence of needle stick injury was 43% among health workers [5]. Since HBV is a bloodborne pathogen, healthcare professionals should be immunized, with the occurrence being about 2 to 10 times higher than in the general population [6]. Improving public understanding of the signs, symptoms, and causes of HBV infection is a critical strategy for preventing disease spread [2]. As a result, various studies have been conducted across the world to assess healthcare students’ knowledge, attitudes, and practices (KAP) about HBV and its related variables [7,8,9,10]. Healthcare students in their clinical years should have sufficient awareness of HBV infection, the modes of transmission, the main symptoms, complications, and protective measures, as it would improve their health and direct them to be more sensitive and conscientious toward themselves and their patients [11,12,13]. There is a need for up-to-date data regarding the level of awareness among healthcare students in Jordan about HBV infection. In this study, which included students from different colleges (medicine, dentistry, nursing, and pharmacy) within various universities across the country, we aimed to determine the KAP levels toward HBV infection among healthcare students and assess the related factors.

## 2. Methodology

A cross-sectional study was conducted. We were able to obtain responses from 2322 participants in our study during the period between March and August 2022. Our target population was healthcare students, who finished the infectious disease course (i.e., 3rd year to 6th year) and were part of one of the following four colleges (medicine, nursing, dentistry, and pharmacy) from any of the six universities in Jordan where a college of medicine was present: Jordan University of Science & Technology, Mut’ah University, University of Jordan, Hashemite University, Al-Balqa Applied University, and Yarmouk University. A participant-driven sampling approach was applied to recruit participants from medical colleges (i.e., medicine, nursing, dentistry, and pharmacy) within different universities. After obtaining ethical approval, we reached out to the students’ representatives for each batch across the universities to facilitate distributing the questionnaire to eligible participants. The questionnaire was sent to all students through messages and posted in each batch’s main official social media groups. These groups are private groups under the supervision of the students’ representative of the concerned batch. 

### 2.1. Questionnaire Administration

An invitation letter was sent to the students who are eligible to participate in the study. The questionnaire was distributed using an online Google Form survey link. After signing the informed consent form, students were asked to complete the questionnaire. It was explicitly mentioned that participation was entirely voluntary and that the individual may quit at any moment without penalty. It was also stated that there would be no direct advantages of participating other than sharing current knowledge. No personally identifiable information was gathered, and participants were asked to provide the necessary information to achieve precise findings.

### 2.2. Measurement Tool

A structured, online-based, Arabic/English, self-administered questionnaire—based on a previously validated instrument—was adopted [14,15]. The Arabic version was completed by two researchers and divided into four sections: the demographics of participants, a knowledge section consisting of (43) questions, an attitudes section with (8) questions, and a practices section with (3) questions; the questionnaire is shown in S1: Questionnaire supplementary file. Before distributing and implementing the questionnaire, a pilot test was conducted with 30 medical students to make sure the questions were understandable and clear.

### 2.3. Ethical Considerations

The study protocol was developed in accordance with the Helsinki Declaration’s ethical standards, and it was evaluated and approved by the Institutional Review Board (IRB) at the University of Jordan in Amman, the Hashemite Kingdom of Jordan, on 25 January 2022 (reference number: 1/2022/2506) in meeting No. 2022/1. Before completing the questionnaire, all participants provided online informed consent. The data were gathered and processed confidentially before being saved on a personal computer that only authors could access.

### 2.4. Statistical Analysis

Data were entered into Microsoft Excel (2016) and then imported into IBM SPSS version 25 (IBM Corp., Armonk, NY, USA) for analysis. For each quantitative and categorical variable, descriptive statistics were computed and expressed as either frequency and percentage or mean and standard deviation (S.D.). Associations between demographic variables, knowledge, attitudes, and practices were analyzed using chi-square tests. Significantly associated variables were included in multivariate regression analysis, and potential confounders were controlled for, to assess the independent effect of each variable. Each true answer received one point. A participant’s score was considered good if they successfully answered more than 70% of the questions in each KAP section. Participants’ scores were considered low if they successfully answered fewer than 70% of the questions in each section. A *p*-value of α < 0.05 and a confidence interval of 95% were set to determine the statistical significance of the reported results.

## 3. Results

### 3.1. Demographics of Survey Participants

The demographic characteristics of the study sample are presented in Table 1. A total of 2322 students responded to the questionnaire. Overall, 67.9% (*n* = 1577) were female, and 32.1% (*n* = 745) were male. The mean age of participants was (22.2 ± 1.7). Further, 26.4% (*n* = 612) of the participants were medical students, 25% (*n* = 581) were nursing students, 22.9% (*n* = 532) were pharmacy students, and 25.7% (*n* = 597) were dental students. As far as students’ year of study, 35.9% (*n* = 833) were in their 3rd year, 23.3% (*n* = 541) were 4th year, 14% (*n* = 324) were in 5th year, and 26.9% (*n* = 624) were in 6th year. Only 10.8% (*n* = 250) took extra courses (outside of their university lectures) about HBV. Further, 1.2% (*n* = 29) were infected with HBV, 3.7% (*n* = 86) had a family member infected with HBV, and 32.3% (*n* = 749) had encountered patients with HBV infection.

### 3.2. Knowledge about Hepatitis B Virus and Its Associated Factors

A summary of the participants’ HBV knowledge is shown in the Supplementary File S2, Table S1. About 86.3% did not know the estimated prevalence of HBV infection in Jordan. Only 14.3% knew that newborns infected with HBV were the highest risk group, and 15.5% knew that most HBV patients in Jordan got infected through mother-to-child transmission at birth. Additionally, 11.8% knew about the high risks of premature death without monitoring and treatment. About two-thirds (69.8%) of the participants knew that HBV could cause liver cirrhosis, liver failure, liver cancer, or premature death.

Most participants claimed that HBV could be transmitted through blood transfusion (94.8%), mother-to-child at birth (80.5%), and unprotected sex (82.6%) and not by sneezing, coughing (60.2%), or handshaking (75.8%). However, (58.9%) still wrongly believed that HBV could be transmitted through sharing utensils and food with an HBV-infected person. Regarding HBV vaccination, (86.6%) of the participants recognized that hepatitis B vaccination could prevent HBV infection. A minority (23.6%) knew newborn vaccination at birth could prevent mother-to-child transmission. Few participants (12.1%) knew that giving the first dose of the hepatitis B immune globulin (HBIG) within 12 h of birth and completing the vaccine doses is the most effective way to protect infants born from HBsAg-positive mothers. Knowledge about needle stick injury was assessed; about half (48.4%) of the participants knew they should dispose of used needles and syringes into a sharp-proof container immediately without recapping. In addition, 32.9% knew they should not recap the needle with two hands to prevent needle stick injury. Participants’ knowledge about hepatitis B diagnosis and management was inadequate. Only 6.1% knew that most patients with HBV are often asymptomatic, and 41.8% knew that the hepatitis B surface antigen (HBsAg) test is the test used to confirm HBV infection. Further, 37.8% knew that the hepatitis B surface antibody (anti-HBs) test is used to identify immunity to hepatitis B, and 6% knew that infants, who were born to HBsAg-positive mothers, should be evaluated for HBsAg after one year of delivery. More than half of the participants knew HBV screening is recommended for pregnant women, HIV patients, men who have sex with men, and family members of the infected patients. About 56.5% were aware that there is no cure for HBV, but there are effective medications to manage and control the disease, while 75.5% wrongly believed that all HBV patients need to receive antiviral treatment, and 48.2% were unaware that treatment of HBV with nucleotide or nucleoside analogs (NAS) is long-term and potentially lifelong.

Participants’ gender, having taken extra HBV courses, being infected with HBV, and having a family history of HBV were not associated with knowledge level, so they were not included in the multivariate logistic regression analysis. See Table 2.

In the multivariate logistic regression analysis, significant associations were found between the level of knowledge and the following variables: age (OR = 1.007, CI = 0.934–1.087, *p* < 0.852); college: medicine (OR = 2.174, CI = 1.706–2.77, *p* < 0.000; ref: Pharmacy), nursing (OR = 1.147, CI = 0.884–1.489, *p* < 0.301; ref: Pharmacy), dentistry (OR = 0.681, CI = 0.531–0.873, *p* < 0.000; ref: Pharmacy); studying year: 4th year (OR = 1.322, CI = 1.019–1.714, *p* < 0.035; ref: 3rd year), 5th year (OR = 2.764, CI = 1.984–3.850, *p* < 0.00; ref: 3rd year), 6th year (OR = 2.974, CI = 2.096–4.218, *p* < 0.00; ref: 3rd year); and whether they had encountered an HBV patient (OR = 1.642, CI = 1.350–1.998, *p* < 0.00). See Table 3.

### 3.3. Attitudes toward Hepatitis B Virus and Its Associated Factors 

The summary of the participants’ correct answers regarding HBV attitudes is shown in Supplementary File S2, Table S2. The majority of participants (82.3%) believed that the HBV vaccine was safe, and 55.5% thought that it was necessary to vaccinate newborns at birth. Almost half (48.1%) were assured in counseling patients on HBV prevention. In addition, 58.9% and 63.4% were confident in ordering laboratory tests to diagnose HBV and to monitor patients with HBV, respectively. However, 29.5% were confident in prescribing medications to treat HBV patients. Hepatitis B stigma among the study participants was high, with 68.5% expressing concerns about casual contact or working in the same office and 77% expressing concerns about eating or sharing food with a person with HBV. The variables related to HBV infection history were not included in the multivariate logistic regression analysis since they were not associated with attitudes. Significant associations were found between attitudes and the following variables: age (OR = 1.051, CI = 0.976–1.133, *p* < 0.189); gender (male: OR = 1.322, CI = 1.100–1.590, *p* = 0.003; ref: female); college: medicine (OR = 1.955, CI = 1.544–2.476, *p* < 0.000; ref: pharmacy), nursing (OR = 1.664, CI = 1.249–2.140, *p* < 0.000; ref: pharmacy), dentistry (OR = 0.876, CI = 0.687–1.113, *p* < 0.274; ref: pharmacy); studying year: 4th year (OR = 1.151, CI = 0.899–1.472, *p* < 0.264; ref: 3rd year), 5th year (OR = 1.223, CI = 0.884–1.692, *p* < 0.225; ref: 3rd year), 6th year (OR = 1.146, CI = 0.814–1.613, *p* < 0.435; ref: 3rd year); HBV patient encounter (OR = 1.440, CI = 1.189–1.746, *p* < 0.00); and whether they had taken extra HBV courses (OR = 1.371, CI = 1.040–1.807, *p* < 0.025).

### 3.4. Practices toward Hepatitis B Virus and Its Associated Factors

The summary of the participants’ correct answers regarding HBV practices is shown in the Supplementary File S2, Table S3. Around 70% of the participants sought to receive the hepatitis B vaccine before starting clinical training at the hospitals, and 45.1% of them sought to receive hepatitis B testing. About 61.6% reported consistently wearing gloves when administering injections or performing medical procedures at hospitals. The variables related to HBV infection history were not included in the multivariate logistic regression analysis since they were not associated with practices. In the multivariate logistic regression analysis, significant associations were found between the level of practices and the following variables: age (OR = 1.046, CI = 0.960–1.140, *p* < 0.300); gender (male: OR = 1.197, CI = 0.974–1.470, *p* = < 0.087; ref: female); college: medicine (OR = 1.656, CI = 1.301–2.109, *p* < 0.000; ref: pharmacy), nursing (OR = 3.977, CI = 3.052–5.183, *p* < 0.000; ref: pharmacy), dentistry (OR = 7.698, CI = 5.876–10.087, *p* < 0.000; ref: pharmacy); studying year: 4th year (OR = 1.011, CI = 0.776–1.317, *p* < 0.934; ref: 3rd year), 5th year (OR = 1.943, CI = 1.354–2.787, *p* < 0.00; ref: 3rd year), 6th year (OR = 1.778, CI = 1.206–2.620, *p* < 0.004; ref: 3rd year); HBV patient encounter (OR = 2.031, CI = 1.634–2.524, *p* < 0.00); and whether they had taken extra HBV courses (OR = 1.437, CI = 1.042–1.981, *p* < 0.027).

### 3.5. Correlation between KAP

Spearman rank correlations revealed significant weak to moderate positive linear correlations between knowledge–attitudes (r = 0.249, *p* < 0.00), knowledge–practice (r = 0.127, *p* < 0.00), and attitudes–practice (r = 0.156, *p* < 0.00). This result reaffirms the relationships between knowledge, attitudes, and practices toward HBV infection, as shown in Table 4.

## 4. Discussion

Promoting awareness is a cornerstone of any strategy to control and prevent infectious diseases. The aim of this cross-national study was to assess the level of KAP among healthcare students toward HBV infection in Jordan. A total of 2322 healthcare students in their clinical years completed the questionnaire. This study revealed that the overall knowledge regarding HBV infection, prevalence, sequelae, diagnosis, transmission, preventive measures, and treatment was low. In our study, around forty percent of healthcare students had a high level of knowledge. This result was similar to a study conducted in India [7]. However, other studies showed a high level of knowledge among healthcare students [8,9]. We have noticed a significant knowledge gap regarding HBV prevalence, sequelae, diagnosis, preventive measures, testing, and interpretation of hepatitis B test results. Approximately less than 15% of the healthcare students were aware of HBV prevalence, the main route of transmission of HBV infection, and who is at higher risk of contracting the infection. Only 6.1% were aware that most HBV patients are without symptoms, about 42% knew that HBsAg is used for the diagnosis of HBV infection, and 56.5% were aware that there is no cure but there are effective medications to manage the disease, as observed in other studies [8,10]. This fact may be attributed to an inadequate virology curriculum and insufficient clinical training in interpreting test results. Scientific knowledge about HBV is essential for healthcare students to minimize the risk of viral transmission during their clinical training. In our study, the level of knowledge about HBV infection was higher among those who studied medicine or had high education level. In addition, healthcare students who encountered patients with HBV infection were more knowledgeable than those who did not. The poor knowledge levels exhibited in our study are alarming, and efforts should be made to explore the reasons behind such poor knowledge and understand the causes.

Generally, attitudes and beliefs toward HBV infection among healthcare students in Jordan were poor, which is in line with studies conducted in Ghana and Cameroon [16,17]. Only 42% of the healthcare students held positive attitudes, a very low percentage in comparison to other studies [7,18]. The stigma of HBV infection is still a significant issue that should be targeted among healthcare students. About 23% of students had concerns about sharing food or utensils with patients having HBV, while 31.5% had concerns about contacting or working with individuals infected with HBV. This was similar to another study done in Jordan among nursing students who were afraid of treating people with hepatitis, as they thought they might be at risk and that people infected with hepatitis should be isolated in a special room [19]. On the other hand, more than half (56.1%) of Saudi dentists were willing to achieve continuity of care for HBsAg-positive patients [10]. This stigma increases the psychological and social burden on patients already suffering from HBV and its medical consequences, which poses significant challenges. We observed that being of the male gender, being in a medical college, having a high level of education, having taken extra courses about HBV infection, and having experience with HBV patient encounters were good predictors of holding better attitudes. Despite the low level of both knowledge and attitudes, healthcare students had satisfactory levels of good practices toward HBV infection. This was similar to other studies conducted in Vietnam and Ghana [14,20]. Furthermore, our results showed that 45.1% of healthcare students reported having an HBV test before starting clinical training at hospitals, while 70.7% of them reported receiving an HBV vaccine, similar to another study done in Jordan among students in dental practices [21]. In addition, the majority (82.3%) of healthcare students believed that the HBV vaccine is safe; this finding is in line with other studies done in Ethiopia and Saudi Arabia, and in contrast to an Italian study done among dentists [8,10,22]. To achieve universal hepatitis B vaccine coverage for all healthcare students, schools must implement programs to evaluate students’ immunity status and provide vaccines to unvaccinated individuals. Only 32.9% knew that recapping needles is incorrect practice and can cause needle stick injury. This explains the high rates of needle stick injury among nursing staff in Jordan, as found in a previous study [19]. These findings emphasize the need for infection control training to improve injection safety practices among students. In our study, we noticed that having more clinical years spent as trainees in hospitals, as well as having encountered HBV patients, was related to higher levels of knowledge, attitudes, and practices achieved.

### Strengths and Limitations

To the best of our knowledge, this study surveyed the biggest sample of healthcare students from the different colleges of Jordan’s universities, thus ensuring a considerable degree of representation and facilitating the extraction of comprehensive data. The study has limitations, such as data being based on participant self-reporting and being unable to be checked. Similarly, estimates of testing and vaccination coverage, as well as HBsAg seroprevalence, relied only on participant response. Because this is a cross-sectional study, establishing a cause-and-effect relationship is challenging. In addition, only quantitative measures and online convenience sampling were used to assess awareness, which are inevitable limitations.

## 5. Conclusions

The findings of this study revealed unsatisfactory knowledge and attitude scores toward HBV infection. However, the level of practice is satisfactory among healthcare students. Students who were from the college of medicine, had higher education levels, or had encountered a patient with HBV infection were shown to possess higher levels of knowledge. Being a male student, from the medical college, who encountered patients with HBV infection or took extra courses about HBV were related to more positive attitude levels. In terms of practices, a high level of practice was shown among students from the college of dentistry, those encountering patients with HBV infection, and those having taken extra courses regarding HBV infection. Finally, it is necessary to implement enlightenment programs to improve healthcare students’ knowledge, attitudes, and practices, particularly in weak areas.

In addition, we recommend that future studies explore students’ HBV test and immunization statuses in terms of their associations with HBV KAP levels, as well as the prevalence of HBV-caused needle stick injury among healthcare providers.

## Figures and Tables

**Table 1:** Demographics of survey participants (*n* = 2322).

Respondents’ Demographics		*n*/Mean	%/SD
Age		22.2	(±1.7)
Gender:	Male	745	32.1%
	Female	1577	67.9%
College:	Medicine	612	26.4%
	Nursing	581	25.0%
	Pharmacy	532	22.9%
	Dentistry	597	25.7%
Studying year:	3rd year	833	35.9%
	4th year	541	23.3%
	5th year	324	14%
	6th year	624	26.9%
Extra courses about HBV:	Yes	250	10.8%
	No	2072	89.2%
History of HBV infection:	Yes	29	1.2%
	No	2293	98.8%
Family member infected with HBV:	Yes	86	3.7%
	No	2236	96.3%
Encountered HBV patient:	Yes	749	32.3%
	No	1573	67.7%

**Table 2:** Association between demographic characteristics of students and knowledge, attitudes, and practices toward HBV (*n* = 2322).

		Level of Knowledge			Level of Attitude			Level of Practice		
		Low (1404)	High (921)	*p*-Value	Low (1355)	High (967)	*p*-Value	Bad (838)	Good (1484)	*p*-Value
Age:		21.9(±)	22.6(±)	0.000	220.1(±)	220.3(±)	0.000	210.9(±)	220.4(±)	0.000
Gender:	Male	470	275	0.063	387	358	0.000	234	511	0.001
	Female	931	646		968	609		604	973	
College:	Medicine	278	334	0.000	298	314	0.000	287	325	0.000
	Nursing	385	196		305	276		168	413	
	Pharmacy	315	217		347	185		294	238	
	Dentistry	423	174		405	192		89	508	
Studying year:	3rd year	608	225	0.000	518	315	0.004	343	490	0.000
	4th year	353	188		303	238		216	325	
	5th year	169	155		199	125		108	216	
	6th year	271	353		335	289		171	453	
Extra courses about HBV:	Yes	148	102	0.698	118	132	0.000	67	183	0.001
	No	1253	819		1237	835		771	1301	
History of HBV infection:	Yes	17	12	0.849	12	17	0.062	9	20	0.568
	No	1384	909		1343	950		829	1464	
Family member infected with HBV:	Yes	50	36	0.671	47	39	0.478	37	49	0.172
	No	1351	885		1308	928		801	1435	
Encountered HBV patient:	Yes	364	385	0.000	369	380	0.000	192	557	0.000
	No	1037	536		986	587		646	927	

**Table 3:** Logistic regression analysis between demographic characteristics of healthcare students and knowledge, attitudes, and practices toward HBV.

Covariates	Knowledge			Attitude			Practice		
	OR	CI	*p*-Value	OR	CI	*p*-Value	OR	CI	*p*-Value
Age:	1.007	0.934–1.087	0.852	1.051	0.976–1.133	0.189	1.046	0.960–1.140	0.300
Gender:	NA								
Male				1.322	1.100–1.590	0.003	1.197	0.974–1.470	0.087
Female				Reference			Reference		
College:									
Pharmacy	Reference			Reference			Reference		
Medicine	2.174	1.706–2.77	0.000	1.955	1.544–2.476	0.000	1.656	1.301–2.109	0.000
Nursing	1.147	0.884–1.489	0.301	1.664	1.249–2.140	0.000	3.977	3.052–5.183	0.000
Dentistry	0.681	0.531–0.873	0.000	0.876	0.687–1.113	0.274	7.698	5.876–10.087	0.000
Studying year:									
3rd year	Reference			Reference			Reference		
4th year	1.322	1.019–1.714	0.035	1.151	0.899–1.472	0.264	1.011	0.776–1.317	0.934
5th year	2.764	1.984–3.850	0.000	1.223	0.884–1.692	0.225	1.943	1.354–2.787	0.000
6th year	2.974	2.096–4.218	0.000	1.146	0.814–1.613	0.435	1.778	1.206–2.620	0.004
Encountered HBV patient:									
Yes	1.642	1.350–1.998	0.000	1.440	1.189–1.746	0.000	2.031	1.634–2.524	0.000
No	Reference			Reference			Reference		
Extra courses about HBV:	NA								
Yes				1.371	1.040–1.807	0.025	1.437	1.042–1.981	0.027
No				Reference			Reference		

**Table 4:** Correlations between knowledge, attitudes, and practice scores.

Variable	Correlation Coefficient	*p*-Value *
Knowledge–attitude	0.249	0.000
Knowledge–practice	0.127	0.000
Attitude–practice	0.156	0.000

* Correlation significant at 0.01 level (2-tailed).

## Data Availability

The data that support the findings of this study are available from the corresponding author (N.A.) upon reasonable request.

## Supplementary Materials

The following supporting information can be downloaded at: https://www.mdpi.com/article/10.3390/ijerph20054348/s1, File S1: Questionnaire; File S2: KAP Correct Answers Tables; Table S1. Knowledge about HBV among healthcare students in Jordan (n = 2322). Table S2. Attitude toward HBV (n = 2322). Table S3. HBV Preventive Practices (n = 2322).
