# Supplementary material for: Knowledge, Attitude, and Practices toward Hepatitis B Infection among Healthcare Students—A Nationwide Cross-Sectional Study in Jordan
Source: Int J Environ Res Public Health. 2023 Feb 28;20(5):4348. doi: 10.3390/ijerph20054348 (PMC10002228; doi:10.3390/ijerph20054348)
Supplement: Supplementary file 1 [file ijerph-20-04348-s001.zip › File S1. Questionnaire.pdf]

English version

Sociodemographic Data:
1- Age (in year):
2-Gender:
a-male
b-female
3- In which college you are:
a- medicine college
b-nursing college
c- Dentistry college
d- Pharmacy college
4- In which year you are:
a- 3rd year
b- 4th year
c- 5th year
d- 6th year
5-Have you taken any extra HBV courses (beside your university lectures, this includes summer courses, seminars/webinars, ...)?
a-Yes
b-No
6-Have you been infected with HBV
a-Yes
b-No
7-Do you have a family member infected with HBV
a-Yes
b-No

1. In your opinion, approximately how many percent of Jordanian population has hepatitis B?
(Please check ONE):
A. 2%
B. 5%
C. 10%
D. 25%
E. I don't know
2. In your opinion, how did most people who have hepatitis B in Jordan got infected?
(Please check ONE):
A. Infected mother to child at birth
B. Unsafe injections
C. Contaminated food or water
D. Unprotected sex
E.I don't know
3. A person is most likely to develop hepatitis B infection after the initial infection at?
(Please check ONE):

A. Newborns
B. Teenagers
C. Middle-age and seniors
D. Age is not a factor
4. In your opinion, HBV can cause which of the followings? (Please check ONE):
A. Liver cirrhosis
B. Liver failure
C. Liver cancer
D. Premature death
E. All of the above
F. I don't know
Question 6-10: How could HBV be transmitted?
5. Shaking hands with a person infected with HBV
A. True
B. False
C. Don't know
6. Having unprotected sex with a person infected with HBV
A. True
B. False
C. Don't know
7. Having blood transfusion
A. True
B. False
C. Don't know
8. Sneezing or coughing
A. True
B. False
C. Don't know
9. From mother to her child at birth
A. True
B. False
C. Don't know
10. Eating with or sharing food and utensils with a person with HBV
A. True
B. False
C. Don't know
Questions 11-15: What can prevent hepatitis B transmission?
11. Clean and cook food thoroughly
A. True
B. False
C. Don't know
12. Provide hepatitis B vaccination to persons with no immunity
A. True
B. False
C. Don't know
13. Do not reuse or share needles/syringes
A. True
B. False

C. Don't know
14. Avoid sharing food and utensils or eating with a person with HBV
A. True
B. False
C. Don't know
15. Use condom
A. True
B. False
C. Don't know
16. What is the best HBV prevention for children whose mother is a hepatitis B carrier or has hepatitis B?
A. Administer HBIG shot
B. Administer HBV Vaccine within first 24 hours of birth
C. Administer the combination of HBIG shot and three doses of VGB Vaccine
D. Don't know
17. Are you confident in consulting patients about preventions for HBV?
A. Yes
B. No
C. Not sure / Don't know
18. In your opinion, who needs to be vaccinated to prevent hepatitis B infection?
A. All healthy and stable newborns
B. Family members of someone who has HBV
C. Sex partner of persons with HBV
D. Healthcare workers without immunity
E. All of the above
F. I don't know
19. Do you think the hepatitis B vaccine is safe (Please check ONE):
A. Very safe
B. Maybe safe
C. I don't know
20. When would you give a healthy and stable baby the first dose of hepatitis B vaccine? (Please check ONE)
A. Within the first 24 hours of birth
B. 1- 7 days old
C. 1 month old
D. I don't know
21. What should a pregnant woman, who has hepatitis B, do to protect the newborn from becoming infected? (Please check ONE)
A. Administer hepatitis B vaccine to the pregnant woman
B. Administer the first dose of hepatitis B vaccine and the HBIG shot within 12 hours of birth then complete the vaccine series
C. Administer the first dose of hepatitis B vaccine and the HBIG shot after 48 hours of birth then complete the vaccine series
D.I don't know

22. Does the University where you are studying require medical students to get

vaccinated against HBV prior to internship at hospital?
A. Yes
B. No
C. Not sure / Don't Know
23. Does the University of Medicine where you are studying require medical students to get tested for HBV prior to internship at the hospital?
A. Yes
B. No
C. Not sure / Don't Know
Question 24-27: As a medical student, which of the followings do you think can help healthcare workers to prevent infection from needlestick injury?
24. Wash hands with soap or disinfectant after each clinical procedure
A. True
B. False
C. Don't know
25. Recap needle with two hands after use and discard immediately in a sharp-proof container
A. True
B. False
C. Don't know
26. Do not recap needle and discard immediately in a sharp-proof container
A. True
B. False
C. Don't know
27. Are there sharp-proof containers at your clinic for disposing needles and sharp objects? (Please check ONE):
A. Always
B. At some places
C. Not available
D. I don't know
28. Do you wear gloves when administering injections to patients? (Please check ONE):
A. Always
B. At some places
C. Not available
D. I don't administer injections to patients

DIAGNOSIS AND MANAGEMENT FOR HBV PATIENTS
Question 29-32: In your opinion, which of the following patient groups would you order hepatitis B screening test even if they have normal level of AST / ALT in liver, or don't have hepatic symptoms?
29. Pregnant women
A. Yes
B. No
C. Don't know
30. Persons infected with HIV
A. Yes
B. No

C. Don't know
31. Men who have sex with men (MSM)
A. Yes
B. No
C. Don't know
32. Family members of hepatitis B patients
A. Yes
B. No
C. Don't know
33. Which single test would you order to confirm that a patient has hepatitis B? (Please check ONE):
A. HBsAg
B. Anti-HBs
C. Anti-HBc
D. HBeAg
34. Which single test would you order to know if a patient has immunity to hepatitis B? (Please check ONE):
A. HBsAg
B. Anti-HBs
C. Anti-HBc
D. Anti-HBe
E. HBeAg

35. In your opinion, what is the symptom most patients with hepatitis B present? (Please check ONE):
A. Headache and fatigue
B. Nausea or vomiting
C. Loss of appetite
D. Jaundice
E. All of the above
F. None, there are usually no symptoms
G. I don't know
36. Which measurement in people with positive HBsAg show the need to order for treatment? (Please check all that apply):
A. ALT level
B. HBV DNA
C. Evaluation of liver cirrhosis
D. The combination of the three above
E. I don't know
37. Which of the following statements is true about HBV treatment? (Please check all that apply):
A. HBV is curable
B. There is no cure, but there are medications effective to manage and control the disease
C. There is no treatment available, but there is herbal medicine that can help to slower the disease progression.
D. I don't know
Question 41-44: What are the goals of HBV Treatment?
38. Sustain inhibition of HBV replication

A. True
B. False
C. Don't know
39. Improve quality of life and prevent progression to liver cirrhosis and liver cancer:
A. True
B. False
C. Don't know
40. Prevent spread of HBV infection in community, including infection from mother to child at birth:
A. True
B. False
C. Don't know
41. Prevent outbreak of HBV:
A. True
B. False
C. Don't know
Question 45-47: In your opinion, what are the rules in HBV treatment?
42. First line drugs are nucleot(s)ide analogues (NAs)
A. True
B. False
C. Don't know
43. Treatment of HBV with NAs is long term, or can be for a life time
A. True
B. False
C. Don't know
44. Patients need to follow / obey the treatment process
A. True
B. False
C. Don't know
45. Do you think that all patients with HBV need to be treated? (Please check ONE):
A. Yes, all patients with HBV should receive treatment as soon as possible
B. Only patients with active liver damage or cirrhosis need to be treated
C. There is no need to treat HBV because there is no cure yet
D. I don't know
46. Which of the following is correct about monitoring HBV patients? (Please check ONE):
A. Only patients with symptoms need to be regularly monitored and screened
B. Only patients who are on HBV treatment need to be regularly monitored and screened
C. All patients with HBV need to be regularly monitored and screened, regardless of treatment indication
E. I don't know
47. Without proper monitoring and treatment, what is the chance a patients would die of complications of hepatitis B? (Please check ONE):
A. Less than 5%
B. 5-10%
C. 15-25%
D. >30-40%%
E. Over 40%

G. I don't know
48. Are you confident in ordering the tests to monitor patients with HBV?
A. Yes
B. No
C. Don't know / Not sure
49. Are you confident in giving prescriptions to patients with HBV?
A. Yes
B. No
C. Don't know / Not sure
50. In your opinion, when should newborns from mothers who have positive HBsAg be evaluated / screened for their possibility of having HBV infection?
A. Right after birth
B. 6 months
C. 12 months
D. 24 months
E. Don't know
51. Are you confident in ordering diagnosis test for patients with HBV?
A. Yes
B. No
C. Don't know / Not sure
52. Would you have any concern having casual contact or working together with a HBV patients in the same office?
A. Yes
B. No
C. Somewhat concern
53. Would you have any concern eating with (sharing food or utensils) with a HBV patient?
A. Yes
B. No
C. Somewhat concern

54. Do you feel confident in ordering HBV Vaccination for newborns

A. Yes
B. No
C. Don't know

Answer to the questionnaire			
Question Number	Answer	Question Number	Answer
Q1	A	Q28	Always
Q2	A	Q29	Yes
Q3	A	Q30	Yes
Q4	E	Q31	Yes
Q5	False	Q32	Yes
Q6	True	Q33	A
Q7	True	Q34	B
Q8	False	Q35	F
Q9	True	Q36	D
Q10	False	Q37	B
Q11	False	Q38	Ture
Q12	True	Q39	Ture
Q13	True	Q40	Ture
Q14	False	Q41	Ture
Q15	True	Q42	Ture
Q16	C	Q43	Ture
Q17	Yes	Q44	True
Q18	E	Q45	B
Q19	A	Q46	C
Q20	A	Q47	C
Q21	C	Q48	Yes
Q22	Yes	Q49	Yes
Q23	Yes	Q50	C
Q24	False	Q51	Yes
Q25	False	Q52	No
Q26	True	Q53	No
Q27	Always	Q54	Yes

Arabic version

النسخة العربية

البيانات العامة الاجتماعية:

العمر بالسنوات:

الجنس:

1- ذكر

2- انثى

أنت لأي كلية تنتمي:

1-كلية الطب

2-كلية التمريض

3-كلية الأسنان

4-كلية الصيدلة

أنت في أي مرحلة دراسية:

1-سنة ثالثة

2-سنة رابعة

3-سنة خامسة

4- سنة سادسة

هل قمت بأخذ دورات أو تدريب اضافي إلى محاضرات الجامعة عن التهاب الكبد الوبائي ب (يشمل ذلك الدورات التعليمية الاضافية ، حضور ندورات) في هذا المجال؟

1-نعم

2-لا

هل أصبت بفيروس التهاب الكبد الوبائي ب

1-نعم

2-لا

هل لديك أحد أفراد أسرتك مصاب بفيروس التهاب الكبد ب؟

1- نعم

2- لا

هل واجهت أي مريض مصاب بالتهاب الكبد ب المزمن خلال دراستك السريرية ؟

1- نعم

2-لا

1. بناء على معلوماتك، كم النسبة المئوية التقريبية من سكان الأردن مصابون بالتهاب الكبد ب ؟
   a. ٢٪
   b. ٥٪
   c. ١٠٪
   d. ٢٥٪
   e. لا أعلم
2. بناء على معلوماتك، ما هي طريقة العدوى الاساسية المسؤولة عن اصابات التهاب الكبد ب المزمن في الأردن؟
   a. من الام لطفلها أثناء الولادة

b. الابر غير الامنة
c. الطعام/ المياه الملوث
d. الجنس غير الامن
e. لا أعلم

3. من المرجح أن يصاب الشخص بعدوى التهاب الكبد ب المزمن بعد الإصابة الأولية بالفايروس في العمر الآتي؟
    a. حديثي الولادة
    b. سن المراهقة
    c. البالغين
    d. لا علاقة له بالعمر
    e. لا اعلم
4. بناء على معلوماتك، يمكن أن يسبب الالتهاب الكبد الوبائي ب أي مما يلي؟
    a. تشمع الكبد
    b. فشل الكبد
    c. سرطان في الكبد
    d. الموت المبكر
    e. جميع ما ذكر
    f. لا اعلم

ما هي وسائل انتقال فايروس التهاب الكبد ب ؟

5. مصافحة شخص مصاب بفيروس التهاب الكبد ب
    a. نعم
    b. لا
    c. لا أعلم
6. ممارسة الجنس غير المحمي مع شخص مصاب بفيروس التهاب الكبد ب
    a. نعم
    b. لا
    c. لا أعلم
7. نقل الدم
    a. نعم
    b. لا
    c. لا أعلم
8. الكحة أو العطس
    a. نعم
    b. لا
    c. لا أعلم
9. من الام للطفل عند الولادة
    a. نعم
    b. لا
    c. لا أعلم
10. الأكل مع أو مشاركة الطعام والأواني مع شخص مصاب بفيروس التهاب الكبد ب
    a. نعم
    b. لا
    c. لا أعلم

ما هي وسائل الوقاية من انتقال التهاب الكبد الوبائي ب؟

11. تنظيف الطعام وطهيه جيدًا
    a. نعم
    b. لا
    c. لا أعلم

12. إعطاء لقاح التهاب الكبد B لشخص ليس لديه مناعة
    a. نعم
    b. لا
    c. لا أعلم

13. عدم إعادة استخدام الإبر والمحاقن أو مشاركتها
    a. نعم
    b. لا
    c. لا أعلم

14. تجنب مشاركة الطعام / الأواني أو تناول الطعام مع شخص مصاب بفيروس التهاب الكبد B
    a. نعم
    b. لا
    c. لا أعلم

15. استخدام الواقي الذكري
    a. نعم
    b. لا
    c. لا أعلم

16. ما هي أفضل طريقة لوقاية الأطفال من أمهاتهم المصابات او الحاملات لالتهاب الكبد الوبائي ب المزمن ؟
    a. اعطاء جرعة أجسام مضادة ضد التهاب الكبد الوبائي ب
    b. إعطاء لقاح فايروس التهاب الكبد الوبائي ب خلال الـ 24 ساعة الأولى من الولادة
    c. إعطاء مزيج من حقنة الاجسام المضادة ضد الفايروس وثلاث جرعات من اللقاح
    d. لا أعلم

17. هل تشعر أنك قادر على تثقيف المرضى حول طرق الوقاية من فيروس التهاب الكبد الوبائي ب؟*
    a. نعم
    b. لا
    c. لا أعلم

18. بناء على معلوماتك، من الذي يحتاج إلى أخذ المطعوم للوقاية من عدوى التهاب الكبد ب؟*
    a. جميع الأطفال حديثي الولادة الذين يتمتعون بصحة جيدة ومستقرة
    b. أفراد عائلة شخص مصاب بفيروس التهاب الكبد ب
    c. الشريك الجنسي للأشخاص المصابين بفيروس التهاب الكبد الوبائي
    d. العاملين في القطاع الصحي بدون مناعة ضده
    e. جميع ما ذكر

f. لا أعلم

19. هل تعتقد ان مطعوم التهاب الكبد الوبائي ب آمن؟
    a. آمن
    b. غير آمن
    c. لا أعلم

20. متى ستعطي الطفل الذي يتمتع بصحة جيدة ومستقر الجرعة الأولى من لقاح التهاب الكبد ب؟*
    a. خلال اول ٢٤ ساعة من الولادة
    b. من ١-٧ ايام من الولادة
    c. عمر الشهر
    d. لا اعلم
21. ما الاجراء الذي يمكن أن تتخذه المرأة الحامل المصابة بالتهاب الكبد ب لحماية المولود الجديد من الإصابة بالعدوى؟
    a. أعط لقاح التهاب الكبد ب للمرأة الحامل
    b. أعط الجرعة الأولى من لقاح التهاب الكبد ب والاجسام المضادة ضده في غضون 12 ساعة من الولادة ثم أكمل سلسلة اللقاح
    c. أعط الجرعة الأولى من لقاح التهاب الكبد ب و الاجسام المضادة ضده بعد 48 ساعة من الولادة ثم أكمل سلسلة اللقاح
    d. لا اعلم

22. هل تطلب الكلية التي تدرس بها من طلابها أخذ اللقاح ضد فيروس التهاب الكبد ب قبل التدريب في المستشفى؟*
    a. نعم
    b. لا
    c. لا أعلم
23. هل تطلب الكلية التي تدرس بها من طلابها عمل فحص فيروس التهاب الكبد ب قبل التدريب في المستشفى؟*
    a. نعم
    b. لا
    c. لا أعلم

أي مما يلي يمكن أن يساعد العاملين في مجال الرعاية الصحية على منع انتقال العدوى عن طريق وخزات الابر؟

24. غسل اليدين بالصابون أو المطهر بعد كل إجراء سريري؟
    a. نعم
    b. لا
    c. لا اتذكر

25. إعادة غلق الإبرة بعد الاستخدام وتخلص منها على الفور في الحاوية المخصصة لها؟
    a. نعم
    b. لا
    c. لا اتذكر

26. عدم إعادة إغلاق الابر والتخلص منها فورا في الحاوية المخصصة لها؟
    a. نعم
    b. لا
    c. لا اتذكر

27. هل توجد حاويات للادوات الحادة في عيادتك للتخلص من الإبر والأدوات الحادة؟

a. متوفرة بشكل دائم
b. في بعض الأماكن
c. غير متوفرة
d. لا اعلم

28. هل ترتدي قفازات طبية عندما تقوم بإعطاء الحقن لمريض ما ؟
a. دائماً
b. في بعض الأماكن
c. غير متوفرة
d. لا اعلم

بناء على معلوماتك ، أي من مجموعات المرضى التالية قد تطلب لهم اختبار فحص التهاب الكبد ب حتى إذا كان لديهم المستوى الطبيعي من انزيمات الكبد AST / ALT ، أو ليس لديهم أعراض تدل على مرض في الكبد

29. الحامل
a. نعم
b. لا
c. لا أعلم

30. الأشخاص المصابين ب بفيروس نقص المناعة
a. نعم
b. لا
c. لا أعلم

31. ممارسة الرجل علاقة جنسية مع رجل آخر
a. نعم
b. لا
c. لا أعلم

32. افراد العائلة المصابين بالتهاب الكبد الوبائي ب
a. نعم
b. لا
c. لا أعلم

33. ما هو الاختبار الوحيد الذي تطلبه للتأكد من إصابة المريض بالتهاب الكبد ب المزمن؟*
a. HBsAg
b. Anti-HBs
c. HBeAg
d. لا أعلم

34. ما الاختبار الوحيد الذي تطلبه لمعرفة ما إذا كان المريض لديه مناعة ضد التهاب الكبد ب؟*
a. HBsAg
b. Anti-HBs
c. Anti-HBc

d. Anti-HBe
e. HBeAg
f. لا أعلم

35. برأيك ، ما هو العرض الذي يعاني منه معظم مرضى التهاب الكبد الوبائي ب المزمن؟
a. الصداع والتعب
b. الغثيان أو القيء
c. فقدان الشهية
d. الاصفرار
e. جميع ما ذكر
f. ليس مما ذكر، لا يظهر عليهم أعراض
g. لا أعلم

36. ما هو المقياس عند الأشخاص المصابين بمستضد HBsAg الإيجابي الذي يُظهر الحاجة إلى طلب العلاج؟
a. مستوى انزيم الكبد ALT
b. الحمض النووي للفيروس
c. تقييم درجة التليف
d. جميع ما سبق
e. لا اعلم

37. أي من العبارات التالية صحيح فيما يتعلق بعلاج الالتهاب الكبد ب؟
a. التهاب الكبد الوبائي ب قابل للشفاء
b. لا يوجد علاج ، ولكن هناك أدوية فعالة لتحسين المرض والسيطرة عليه
c. لا يوجد علاج متاح ، ولكن هناك أدوية عشبية يمكن أن تساعد في إبطاء تقدم المرض.
d. لا أعلم

ما هي أهداف علاج التهاب الكبد ب المزمن؟

38. الحفاظ على تثبيط تكاثر فايروس التهاب الكبد الوبائي ب
a. نعم
b. لا
c. لا أعلم

39. تحسين نوعية الحياة ومنع التطور إلى تليف الكبد وسرطان الكبد
a. نعم
b. لا
c. لا أعلم

40. منع انتشار عدوى فيروس التهاب الكبد ب في المجتمع ، بما في ذلك العدوى من الأم إلى الطفل عند الولادة
a. نعم
b. لا
c. لا أعلم

41. منع تفشي فيروس التهاب الكبد الوبائي ب
a. نعم
b. لا
c. لا أعلم

برأيك، ما هي الأساسيات في علاج التهاب الكبد ب؟

42. علاج الخط الأول هي نظائر النيوكليوتيدات
a. نعم
b. لا
c. لا أعلم

43. علاج الالتهاب الكبدي الوبائي المزمن عن طريق نظائر النيوكليوتيدات طويل الأمد، ويمكن أن يستمر مدى الحياة
a. نعم
b. لا
c. لا أعلم

44. يحتاج المرضى لمتابعة / الانصياع لعملية العلاج
a. نعم
b. لا
c. لا أعلم

45. هل تعتقد أن جميع مرضى الالتهاب الكبد ب المزمن يحتاجون إلى العلاج؟
a. نعم ، يجب أن يتلقى جميع مرضى التهاب الكبد الوبائي المزمن العلاج في أسرع وقت ممكن
b. فقط المرضى الذين يعانون من التهاب الكبد النشط أو تليف الكبد هم الذين يحتاجون إلى العلاج
c. ليست هناك حاجة لعلاج الالتهاب الكبد ب لأنه لا يوجد علاج حتى الآن
d. لا أعلم

46. أي مما يلي هو الصحيح فيما يتعلق بمراقبة مرضى التهاب الكبد ب؟
a. فقط المرضى الذين يعانون من الأعراض يحتاجون إلى المراقبة والفحص المنتظم
b. فقط المرضى الذين يخضعون لعلاج التهاب الكبد ب هم الذين يحتاجون إلى المراقبة والفحص بانتظام
c. يجب مراقبة وفحص جميع المرضى المصابين بفيروس التهاب الكبد ب بانتظام، بغض النظر عن حاجتهم للعلاج
d. لا أعلم

47. بدون المراقبة والعلاج المناسبين ، ما هي فرصة وفاة المرضى بسبب مضاعفات التهاب الكبد ب المزمن؟*
a. أقل من 5%
b. 5-10%
c. 15-25%
d. >30-40%%
e. لا اعلم

48. برأيك ، متى يجب تقييم / فحص الأطفال حديثي الولادة من الأمهات اللواتي لديهن فحص HBsAg إيجابي، لإمكانية الإصابة بعدوى فايروس التهاب الكبد الوبائي ب ؟
a. بعد الولادة مباشرة
b. على عمر ٦ شهور
c. على عمر ٦ شهور
d. على عمر ٦ شهور
e. لا اعلم

49. هل لديك الثقة لطلب اختبار التشخيص لمرضى التهاب الكبد الوبائي ب المزمن؟
a. نعم
b. لا
c. لا أعلم

50. هل تشعر بالثقة عند طلب الفحوصات لمراقبة مرضى التهاب الكبد الوبائي ب المزمن؟*

a. نعم
b. لا
c. لا أعلم

51. هل لديك الثقة لكتابة وصفة طبية لمرضى التهاب الكبد الوبائي ب المزمن؟*
a. نعم
b. لا
c. لا أعلم

52. هل لديك أي قلق من الاتصال الاعتيادي أو العمل مع مرضى التهاب الكبد الوبائي ب المزمن في نفس المكتب؟*
a. نعم
b. لا
c. ربما

53. هل لديك أي قلق من تناول الطعام (مشاركة الطعام أو الأواني) مع مريض التهاب الكبد ب المزمن؟*
a. نعم
b. لا
c. ربما

54. هل لديك الثقة لطلب إعطاء اللقاح ضد فايروس التهاب الكبد الوبائي ب لحديثي الولادة؟
a. نعم
b. لا
c. لا اعلم

Answer to the questionnaire			
Question Number	Answer	Question Number	Answer
Q1	A	Q28	دائما
Q2	A	Q29	نعم
Q3	A	Q30	نعم
Q4	E	Q31	نعم
Q5	لا	Q32	نعم
Q6	نعم	Q33	A
Q7	نعم	Q34	B
Q8	لا	Q35	F
Q9	نعم	Q36	D
Q10	لا	Q37	B
Q11	لا	Q38	نعم
Q12	نعم	Q39	نعم
Q13	نعم	Q40	نعم
Q14	لا	Q41	نعم
Q15	نعم	Q42	نعم
Q16	C	Q43	نعم
Q17	نعم	Q44	نعم
Q18	E	Q45	B
Q19	A	Q46	C

Q20	A	Q47	C
Q21	C	Q48	نعم
Q22	نعم	Q49	نعم
Q23	نعم	Q50	C
Q24	لا	Q51	نعم
Q25	لا	Q52	لا
Q26	نعم	Q53	لا
Q27	دائما	Q54	نعم
