# Supplementary material for: Knowledge, Attitude, and Practices toward Hepatitis B Infection among Healthcare Students—A Nationwide Cross-Sectional Study in Jordan
Source: Int J Environ Res Public Health. 2023 Feb 28;20(5):4348. doi: 10.3390/ijerph20054348 (PMC10002228; doi:10.3390/ijerph20054348)
Supplement: Supplementary file 1 [file ijerph-20-04348-s001.zip › File S2. KAP Correct Answers Tables..pdf]

Participants’ correct answers tables

Knowledge

Table S1. Knowledge about HBV among healthcare students in Jordan (n = 2322).

Questions	Correct answers	
	n	%
Prevalence and Sequelae		
1. What percentage of the Jordanian population has chronic hepatitis B (HBV)?	317	13.7%
2. How did most people who have HBV in Jordan get infected?	360	15.5%
3. Which age group is most likely to develop HBV after the initial infection?	333	14.3%
4.What are the consequences of chronic hepatitis B?	1620	69.8%
47. Without proper monitoring and treatment, what is the chance a patient would die of HBV complications?	275	11.8%
Transmission Routes		
5. Can hepatitis B be transmitted through handshake?	1761	75.8%
6. Can hepatitis B be transmitted through unprotected sex?	1919	82.6%
7. Can hepatitis B be transmitted through blood transfusion?	2202	94.8%
8. Can hepatitis B be transmitted through sneezing or coughing?	1397	60.2%
9. Can hepatitis B be transmitted through from mother to child at birth?	1869	80.5%
10. Can hepatitis B be transmitted through sharing food or utensils?	955	41.1%
Prevention Measures		
11. Can cleaning and cooking food thoroughly prevent HBV transmission?	833	35.9%
12. Can the hepatitis B vaccine prevent HBV transmission?	2012	86.6%
13. Can HBV transmission be prevented by not reusing or sharing needles/syringes?	2165	93.2%
14. Can HBV transmission be prevented by avoid sharing food/utensils or eating with a person with chronic HBV?	777	33.5%
15. Can using a condom prevent HBV transmission?	1791	77.1%
16.What is the most effective preventive measure for infants born to mothers with chronic HBsAg?	653	28.1%
18. Who needs the hepatitis B vaccine?	1761	75.8%
21. Prevention of mother-to-child transmission	548	23.6%
20. The first dose of hepatitis B vaccine for baby	280	12.1%
27. Is it necessary to have sharp-proof containers at clinics for disposing of needles and sharp objects?	1467	63.2%
What would you do to prevent needle-stick injury?		
24. Wash hands with soap or disinfectant after each clinical procedure?	292	12.6%
25. Recap needle with two hands after use and discard immediately in a sharp-proof container	763	32.9%
26. Do not recap needle and discard immediately in a sharp-proof container	1124	48.4%
Diagnosis and Treatment		
35. What is the symptom most patients with chronic hepatitis B present with?	142	6.1%
33. Serum HBsAg test for identification of patients infected with hepatitis B virus	971	41.8%
34. What test should be used to identify immunity against the hepatitis B virus?	877	37.8%
50.When should infants born to mothers with HBV be evaluated for HBsAg status?	140	6%

Who should be tested for hepatitis B?		
29. Pregnant women should be tested for hepatitis B	1541	66.4%
30. HIV-infected people should be tested for hepatitis B	1934	83.3%
31. Men who have sex with men (MSM) should be tested for hepatitis B	1581	68.1%
32. Family members of those who have hepatitis B should be tested for hepatitis B	1969	84.8%
Treatment		
36. What are the criteria for indicating treatment in patients with HBV?	787	33.9%
37. There is no cure, but there are effective medications to manage and control the disease?	1312	56.5%
What are the treatment goals for HBV patients?		
38. Inhibit the replication of the hepatitis B virus	1969	84.8%
39. Prevent disease progression of disease, particularly liver cirrhosis and liver cancer	2092	90.1%
40. Prevent mother-to-child transmission (MTCT)	2036	87.7%
41. Prevent flare of hepatitis B	2022	87.1%
42. Is it true that (NAs) are a recommended first-line treatment for HBV?	1092	47.0%
43. Is treatment of HBV with NAs long term, possibly even lifetime?	1120	48.2%
44. Do patients need to strictly adhere to the treatment of HBV?	1724	74.2%
45. Do you think that all patients with chronic HBV need to be treated immediately?	570	24.5%
46. Should all HBV patients be monitored and tested regardless of treatment status?	1486	64%

Abbreviations: HBV: hepatitis B virus; MSM: men who have sex with men; MTCT: mother-to-child transmission; NAs: Nucleotide Analogs.

Attitude

Table S2. Attitude toward HBV (n = 2322).

Questions	Answered yes	
	n	%
17. Are you confident in counseling patients about prevention of HBV?	1118	48.1%
19. Do you think that the hepatitis B vaccine is safe?	1912	82.3%
54. Do you feel confident in ordering HBV Vaccination for newborns?	1289	55.5%
48. Are you confident in ordering laboratory tests to monitor HBV patients?	1473	63.4%
49. Are you confident in prescribing treatment for a patient with chronic hepatitis B?	684	29.5%
51. Are you confident in ordering diagnosis tests for patients with chronic HBV?	1367	58.9%
52. Would you have any concerns having casual contact or working together with a chronic HBV patient in the same office?	731	31.5%
53. Would you have any concerns sharing food or utensils with a HBV?	535	23%

Practices

Table S3. HBV Preventive Practices (n = 2322).

Questions	Answered yes	
	n	%
22. Did you get the hepatitis B vaccine before entering practicum at teaching hospitals?	1642	70.7%
23. Did you get tested for HBV before entering practicum at teaching hospitals?	1047	45.1%
28. Do you consistently wear gloves when administrating injections or performing medical procedures to patients?	1430	61.6%
